# Pharmacological evidence of bradykinin regeneration from extended sequences that behave as peptidase–activated B_2_ receptor agonists

**DOI:** 10.3389/fphar.2014.00032

**Published:** 2014-03-07

**Authors:** Xavier Charest-Morin, Caroline Roy, Émile-Jacques Fortin, Johanne Bouthillier, François Marceau

**Affiliations:** Department of Microbiology and Infectious Disease and Immunology, Université Laval and Centre de Recherche en Rhumatologie et Immunologie–Centre Hospitalier Universitaire de QuébecQuébec, QC, Canada

**Keywords:** angiotensin converting enzyme, arginine carboxypeptidases, bradykinin, vascular smooth muscle, B_2_ receptors for bradykinin

## Abstract

While bradykinin (BK) is known to be degraded by angiotensin converting enzyme (ACE), we have recently discovered that Met-Lys-BK-Ser-Ser is paradoxically activated by ACE. We designed and evaluated additional “prodrug” peptides extended around the BK sequence as potential ligands that could be locally activated by vascular or blood plasma peptidases. BK regeneration was estimated using the contractility of the human umbilical vein as model of vascular functions mediated by endogenous B_2_ receptors (B_2_Rs) and the endocytosis of the fusion protein B_2_R-green fluorescent protein (B_2_R-GFP) expressed in Human Embryonic Kidney 293 cells. Of three BK sequences extended by a C-terminal dipeptide, BK-His-Leu had the most desirable profile, exhibiting little direct affinity for the receptor but a significant one for ACE (as shown by competition of [^3^H]BK binding to B_2_R-GFP or of [^3^H]enalaprilat to recombinant ACE, respectively). The potency of the contractile effect of this analog on the vein was reduced 18-fold by the ACE inhibitor enalaprilat, pharmacologically evidencing BK regeneration *in situ*. BK-Arg, a potential substrate of arginine carboxypeptidases, had a low affinity for B_2_Rs and its potency as a contractile agent was reduced 15-fold by tissue treatment with an inhibitor of these enzymes, Plummer’s inhibitor. B_2_R-GFP internalization in response to 100 nM of the extended peptides recapitulated these findings, as enalaprilat selectively inhibited the effect of BK-His-Leu and Plummer’s inhibitor, that of BK-Arg. The two peptidase inhibitors did not affect BK-induced effects in either assay. The novel C-terminally extended BKs had no or very little affinity for the kinin B_1_ receptor (competition of [^3^H]Lys-des-Arg^9^-BK binding). The feasibility of peptidase-activated B_2_R agonists is illustrated by C-terminal extensions of the BK sequence.

## INTRODUCTION

Angiotensin converting enzyme (ACE; ACE1) is an ectopeptidase expressed by vascular endothelial cells and specific renal epithelial cells; a soluble form in blood plasma also exists since a fraction of the enzyme is cleaved from the endothelial surface. ACE is the molecular target of an important class of drugs, the ACE inhibitors, widely used in the therapy of cardiovascular and renal diseases (Izzo and Weir, 2011). Blocking the metallopeptidase ACE inhibits the conversion of the inactive peptide angiotensin (Ang) I to the active pressor Ang II via the removal of the C-terminaldipeptide His-Leu from the substrate. However, ACE has other peptide substrates that are rather inactivated by the removal of a C-terminal dipeptide, bradykinin (BK) being a prominent example. Thus, ACE (kininase II) inhibition has the potential to potentiate BK effects *in vivo*, especially at the level of preformed and widely expressed B_2_ receptors (B_2_Rs; Leeb-Lundberg et al., 2005). There is limited clinical evidence that BK participates to the anti-hypertensive and other actions of ACE inhibitors as variable fractions of their effects are abated by the specific B_2_R antagonist icatibant (Gainer et al., 1998; Squire et al., 2000; Pretorius et al., 2003).

If endogenous BK, especially via its effect on endothelium-mediated vasorelaxation, has beneficial cardiovascular benefits, one could wonder whether the administration of the peptide or of an analog would be clinically feasible in view of the inflammatory, algogenic, and diarrhea-promoting effect of BK (Manning et al., 1982; Duchene and Bader, 2010). There are few preclinical models where this has been attempted, but they met with apparent success. Thus, in rodents, the administration of peptidase-resistant agonists of the B_2_R improved pulmonary hypertension and its cardiac complication and, following myocardial infarction, reduced the extent of tissue damage and improved cardiac function with beneficial effects on tissue remodeling (Taraseviciene-Stewart et al., 2005; Marketou et al., 2010; Potier et al., 2013). All these effects are postulated to stem from BK-induced endothelium-mediated vasodilation. An application of a B_2_R agonist that deliberately exploited the pro-inflammatory effect of BK was the infusion of labradimil, a BK analog protected against ACE, to temporarily open the blood–brain barrier (a potential adjuvant of the chemotherapy of brain tumors; Packer et al., 2005). Any major vascular leakage or extravascular effects (such as the stimulation of afferent nerve terminals or epithelial cells) could be the source of side effects that would preclude the development of B_2_R agonists as drugs.

Met-Lys-BK-Ser-Ser was recently identified as a kinin produced from the cleavage of high molecular weight kininogen by the neutrophil leukocyte protease PR3 (Kahn et al., 2009). As the vascular pharmacology of this peptide was further explored, it was found that ACE paradoxically activates it (Gera et al., 2011). The Ser–Ser C-terminal extension, presumably removed by ACE, drastically decreases the peptide’s affinity at the B_2_R, while potential reaction products Met-Lys-BK, Lys-BK, and BK are all known high affinity agonists of the B_2_R. This discovery may inspire a “prodrug” strategy where a therapeutic B_2_R agonist would be activated only at the level of vascular endothelial cells, where the circulatory benefits are generated (Hornig et al., 1997).

**FIGURE 1:**
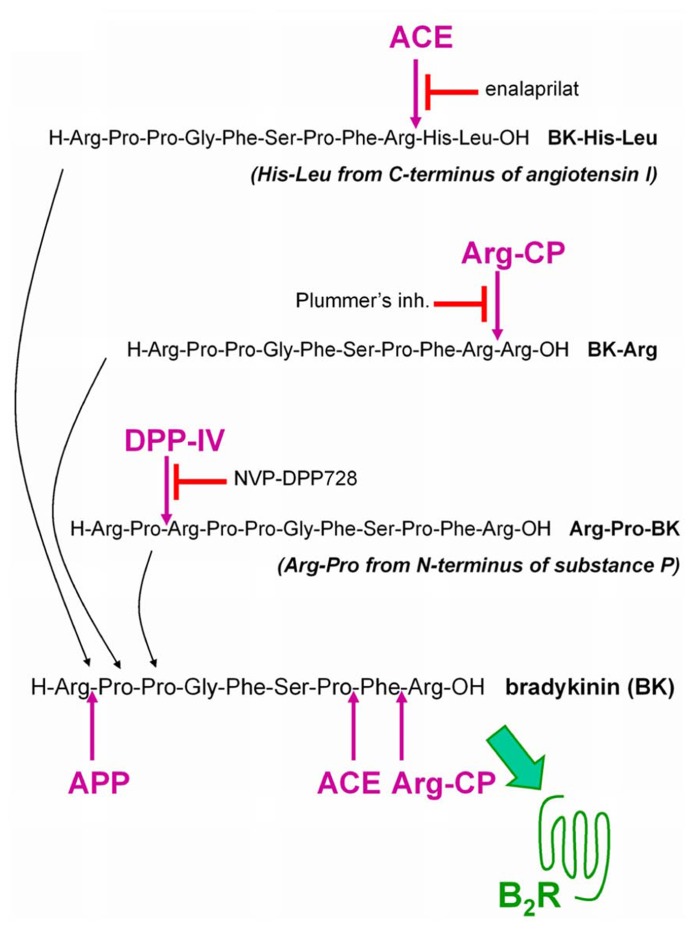
**Extended BK sequences as potential “prodrug” agonists of the B_**2**_R activated by peptidases.** BK is itself degraded by several peptidases that terminate its signaling at B_2_Rs. ACE: angiotensin converting enzyme; APP: aminopeptidase P; Arg-CP: arginine carboxypeptidases; DPP IV: dipeptidyl peptidase-4.

This research program seeks to define a new cardiovascular drug class, the BK B_2_R agonists, that will stimulate the most desirable effects of endothelial B_2_Rs in intensive care situations where an intravenous line is available (unstable angina, myocardial infarction, perhaps decompensated congestive heart failure) and possibly, in more chronic ailments (e.g., pulmonary hypertension). Inspired by the discovery of the ACE-mediated activation of Met-Lys-BK-Ser-Ser, a possible approach to develop such a new class of drugs is to exploit resident vascular peptidases in a prodrug strategy. We designed and evaluated “prodrug” peptides extended around the BK sequence as potential ligands of low potency at the B_2_R, but that could be activated by vascular peptidases. Starting with BK extended by dipeptides as ACE substrates, the concept has been extended to other potential substrates for alternate peptidases present in the vasculature: dipeptidyl peptidase IV (DPP IV; Shah et al., 2011) and arginine carboxypeptidases (Arg-CP; Sangsree et al., 2003; **Figure 1**).

## MATERIALS AND METHODS

### RADIOLIGAND BINDING COMPETITION ASSAYS

Affinity for the B_2_R was evaluated using a radioligand binding competition assay performed at 0^°^C in the presence of peptidase inhibitors that included captopril (Houle et al., 2000). Briefly, the binding of 3 nM [^3^H]BK (Perkin Elmer Life Sciences; 90 Ci/mmol) to adherent intact Human Embryonic Kidney (HEK) 293 cells stably expressing the fusion protein rabbit B_2_R-green fluorescent protein (B_2_R-GFP) was applied to construct binding competition curves for a series of unlabeled peptides. Other HEK 293a cells that transiently expressed human recombinant ACE were used to perform a [^3^H]enalaprilat binding assay as described (Koumbadinga et al., 2010; the peACE vector was a gift from Prof. P. Corvol, Paris, France). A 2 nM concentration of [^3^H]enalaprilat was used to generate binding competition curves for BK and specific analog, as the ACE substrate BK effectively displaces this radioligand (Koumbadinga et al., 2010). It was verified that novel analogs have no affinity at the kinin B_1_ receptor (B_1_R) in a competition assay with the binding of 1 nM [^3^H]Lys-des-Arg^9^-BK (Perkin Elmer Life Sciences; 88 Ci/mmol) to HEK 293a cells transiently expressing the human B_1_R (B_1_R-FLAG vector, Morissette et al., 2008; binding assay as in Gera et al., 2011).

### VASCULAR SMOOTH MUSCLE CONTRACTILITY ASSAY

The institutional research ethics board (CHU de Québec) approved the anonymous use of human umbilical cord segments obtained after elective cesarean section deliveries. Informed consent was obtained from mothers. Umbilical vein rings, used as a contractile bioassay for the BK B_2_R, were prepared and suspended in organ baths and submitted to equilibration in Krebs’ solution as described (Marceau et al., 1994; Gera et al., 2011). The vascular preparation was used to assess the effect of the peptidase inhibitors (introduced 30 min before the agonist) on the apparent potency of BK and related peptides. The full cumulative concentration-effect curves were recorded for each peptide; a large concentration of BK (9.4 μM) was added to record the maximal contractile effect mediated by the B_2_Rs for low potency agonists. Tissues were used only once and discarded; controls curves were obtained from other vascular rings from the same vein.

### DESIGN OF C-TERMINALLY EXTENDED BRADYKININ ANALOGS: ACE SUBSTRATES

BK-Ser-Tyr, BK-His-Leu, BK-Ala-Pro, Arg-Pro-BK, and BK-Arg were custom synthesized by Peptide 2.0 Inc. (Chantilly, VA, USA) via standard solid-phase methodology and provided as >98.8% pure reagents (mass spectroscopy and HPLC analyses). The aim of the design of the three first peptides was to introduce two amino acids that would be easily and selectively cleaved by the carboxydipeptidase ACE, thus restoring BK, the minimal sequence for high-affinity B_2_R activation. At the same time, the C-terminal addition would ideally decrease the affinity of the 11-mers for the B_2_R relative to that of the nonapeptide BK, thus making the reaction with ACE a functional activation one, analogous to the effect of ACE on Ang I.

The first design, BK-Ser-Tyr, was inspired from a study of peptides from fish muscle hydrolysate: this empirical approach identified a tetrapeptide ending with Ser-Tyr as the most efficient competitor of ACE enzymatic activity (Balti et al., 2010). The Ser–Ser C-terminal dipeptide, also found in Met-Lys-BK-Ser-Ser (Gera et al., 2011), was comparatively less efficacious as an ACE competitor (Balti et al., 2010). Therefore, it was hoped that BK-Ser-Tyr would compare favorably with Met-Lys-BK-Ser-Ser as a latent B_2_R agonist activated by ACE. BK-His-Leu included the C-terminal dipeptide His-Leu that is removed from Ang I by ACE. The extension in BK-Ala-Pro is inspired from the structure of captopril, a high affinity ACE inhibitor that mimics a dipeptide. In addition, hippuryl-Ala-Pro is an ACE substrate more efficient (higher k_cat_/K_m_) than hippuryl-His-Leu or Ang I itself (Michaud et al., 1997). Of note, the tetrapeptide Gly-Asp-Ala-Pro was the second best competitor of ACE in the fish protein hydrolysate mentioned above (Balti et al., 2010), further supporting the hypothesis that BK-Ala-Pro has affinity for ACE.

### DESIGN OF OTHER BRADYKININ ANALOGS

Arg-Pro-BK (**Figure 1**) was designed by introducing the N-terminal dipeptide of substance P, a known substrate of DPP IV (Lambeir et al., 2003), at the N-terminus of BK. X-Pro dipeptides (positions P_2_ and P_1_ relative to the sessile bond) are effectively removed from the N-terminus of several peptides by this serine peptidase, but intact BK is not a significant DPP IV substrate due to the presence of Pro^3^ at the P_1_’ position (Lambeir et al., 2003). A potent and highly selective inhibitor of DPP IV, NVP-DPP728 (Hughes et al., 1999), was exploited in the pharmacological evaluation of Arg-Pro-BK.

BK-Arg was designed as a potential substrate of Arg-CP, of which Plummer’s inhibitor (mercaptomethyl-3-guanidinoethyl-thiopropanoic acid; Calbiochem, La Jolla, CA, USA; Plummer and Ryan, 1981) is a selective inhibitor. Arg-CP generate des-Arg^9^-kinins that are selective agonists of B_1_Rs; however, des-Arg^9^-BK has virtually no affinity for the B_2_R and only a weak affinity for the human form of the B_1_R (Leeb-Lundberg et al., 2005) and any activation of BK-Arg in the applied bioassays must proceed via the generation of intact BK.

### OTHER DRUGS

Bradykinin was purchased from Bachem (Torrance, CA, USA). Enalaprilat dehydrate was from Kemprotec Ltd. (Maltby, Middlesbrough, UK) and NVP-DPP728 (1-[[[2-[(5-cyanopyridin-2-yl)amino]ethyl]amino]acetyl]-2-cyano-(S)-pyrrolidine), from Tocris Bioscience (Bristol, UK). The other drugs were from Sigma-Aldrich (St. Louis, MO, USA).

### EFFECT OF AGENTS ON B_2_R-GFP CYCLING

Human Embryonic Kidney 293 cells stably expressing B_2_R-GFP at the level of their plasma membrane exhibit BK-induced endosomal internalization of the fluorescent receptor, maximal 30 min after stimulation but with gradual recycling to the cell surface in 1–3 h (Bachvarov et al., 2001; Charest-Morin et al., 2013b). A microscopic assay of extended BK analogs was based on this system and on the fact that the culture medium containing 10% heat-inactivated fetal bovine serum contains the active soluble form of ACE that remains the main BK inactivation pathway in the system (Bachvarov et al., 2001). Arg-CP(s) sensitive to Plummer’s inhibitor are also found in human plasma and inflammatory synovial fluid that contains serum proteins (Chercuitte et al., 1987), suggesting that serum-containing culture medium may contain this enzymatic activity. HEK 293 cells stably expressing B_2_R-GFP were observed in epifluorescence microscopy at a 1000× magnification and photographed using an Olympus BX51 microscope coupled to a CoolSnap HQ digital camera (filters for GFP and fluorescein: excitation 460–500 nm, emission 510–560 nm, objective lens 100× oil UPlanApo, Olympus). The experiments were based on BK or BK analog stimulation of different durations to monitor either the endocytosis of B_2_R-GFP (30 min) or their recycling (3 h), based on previous time course findings.

### DATA ANALYSIS

Results are presented as mean ± SEM. Radioligand binding data were fitted by non-linear regression to a one-site competition equation using a least-square method (Prism 4.0, GraphPad Software Inc., San Diego, CA, USA) and IC_50_ values calculated from this procedure. The same computer program was used to draw concentration-effect curves (least square fitting of sigmoidal dose-response equation with variable slope) and to derive contractile EC_50_ values. The qualitative effect of treatments on the distribution of the B_2_R-GFP fluorescent protein (either located at the plasma membrane or submitted to internalization) was evaluated for each HEK 293 cell in large microphotographic records and proportion of affected cells were compared using the χ^2^ statistics to determine drug effect on the subcellular receptor distribution.

## RESULTS

### POTENTIAL ACE SUBSTRATES

**FIGURE 2:**
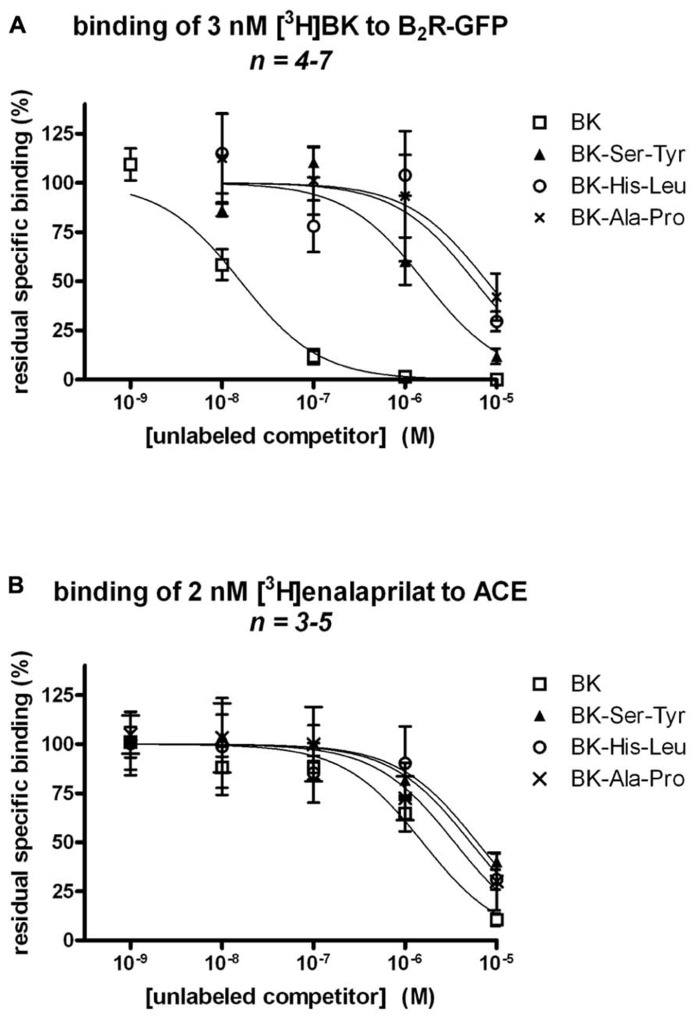
**Competition of the binding of two radioligands at their respective recombinant molecular target by BK or three C-terminally extended analogs developed as potential ACE substrates. (A)** Competition of [^3^H]BK (3 nM) binding to stably expressed B_2_R-GFP in HEK 293 cells. **(B)** Competition of [^3^H]enalaprilat (2 nM) binding to transiently expressed human somatic ACE. Results are expressed as the residual specific bindings (means ± SE of *n* duplicate determinations).

BK-Ser-Tyr, BK-His-Leu, and BK-Ala-Pro exhibited a very low affinity (IC_50_ values 102-, 363-, and 593-fold greater than that of unlabeled BK, respectively) for recombinant B_2_R-GFP, as assessed by the binding competition of [^3^H]BK (**Figure 2A**; numerical values in **Table 1**). This assay was performed on ice in the presence of captopril (see Materials and Methods), thus in the absence of metabolic interference from ACE. By contrast, the affinities of the four peptides for human recombinant ACE determined by the binding competition of [^3^H]enalaprilat was less divergent, the three extended BK analogs exhibiting a 3.94-, 3.33-, and 2.23-fold lesser affinity vs. BK for the peptidase, respectively (**Figure 2B**; **Table 1**).

**Table 1:** Parameters derived from radioligand binding competition assays.

Agonist	[^3^H]BK binding to B_2_R-GFP log(IC_50_) ± SE	[^3^H]enalaprilat binding to ACE log(IC_50_) ± SE	[^3^H]Lys-des-Arg^9^-BK binding to B_1_R-FLAG log(IC_50_) ± SE
BK	-7.80 ± 0.10	-5.81 ± 0.12	>-5
BK-Ser-Tyr	-5.79 ± 0.13	-5.21 ± 0.15	>-5
BK-His-Leu	-5.24 ± 0.20	-5.28 ± 0.22	>-5
BK-Ala-Pro	-5.11 ± 0.16	-5.46 ± 0.11	>-5
Arg-Pro-BK	-7.61 ± 0.09	–	–
BK-Arg	-6.34 ± 0.11	–	-5.12 ± 0.10
Lys-des-Arg^9^-BK	–	–	-8.98 ± 0.05

**FIGURE 3:**
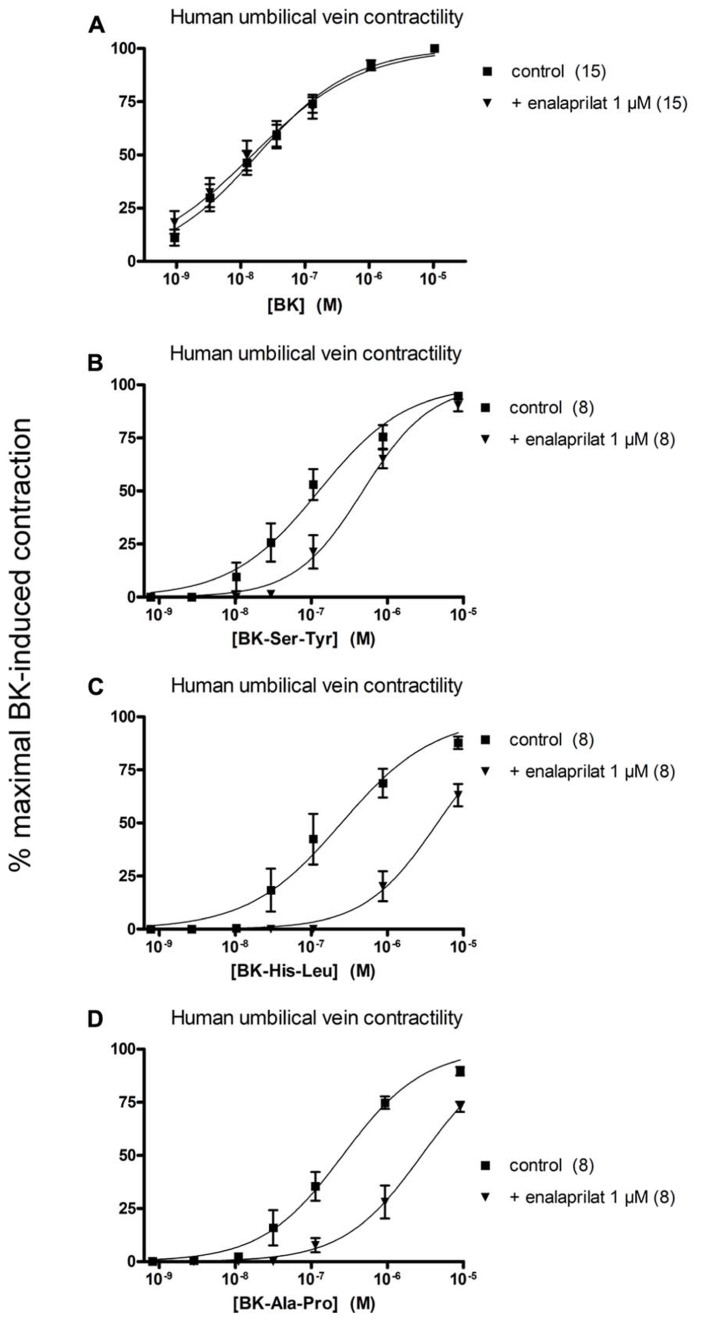
**Contractility studies in the human isolated umbilical vein, a bioassay for the B_**2**_ receptors, for BK and three C-terminally extended analogs that are potential ACE substrates. (A)** Cumulative concentration-effect curve for BK as modified by the ACE inhibitor enalaprilat (1 μM). **(B,C)** Cumulative concentration-effect curve for BK-Ser-Tyr (B), BK-His-Leu **(C)** and BK-Ala-Pro **(D)** as modified by the same drug. The control curves were constructed in the presence of the DMSO vehicle of enalaprilat. Separate tissues from the same individuals were used as controls. Values are means ± SE of the number of replicates indicated between parentheses.

**FIGURE 4:**
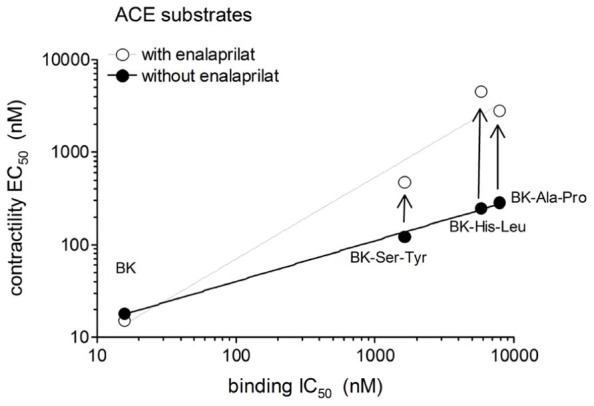
**Relationship between the IC_50_ values obtained using the [^3^H]BK binding competition assay and the contractility EC_**50**_ values of BK analogs that have affinity for ACE as a function of the presence of the ACE inhibitor enalaprilat in the contractility assay**.

The human isolated umbilical vein is a contractile bioassay for the B_2_Rs and this tissue does not exhibit endothelium-dependent vasorelaxation. The three C-terminally extended BK sequences act as contractile agents in the vein (**Figure 3**; parameters calculated from the cumulative concentration-effect curves in **Table 2**). BK-Ser-Tyr, BK-His-Leu, and BK-Ala-Pro were 6.9-, 13.5-, and 14.1-fold less potent than BK, respectively. However, the full blockade of ACE in paired tissues (enalaprilat 1 μM) had contrasting effects on these agents: while the apparent potency of BK was not changed, that of BK-Ser-Tyr, BK-His-Leu of BK-Ala-Pro was decreased 3.9-, 18.3-, and 9.8-fold, respectively. These results support the assumption of a metabolic activation by ACE of latent B_2_R agonists, especially for BK-His-Leu and BK-Ala-Pro. The lack of effect of ACE inhibitors on the apparent potency of BK has been previously reported in this preparation (Marceau et al., 1994; Gobeil et al., 1996; Bawolak et al., 2007). In the contractility assay, the gain of function mediated by endogenous ACE is apparent as a steeper linear regression in the graph representing contractility EC_50_ as a function of binding IC_50_ (**Figure 4**).

### POTENTIAL DPP IV AND ARGININE CARBOXYPEPTIDASE SUBSTRATES

**FIGURE 5:**
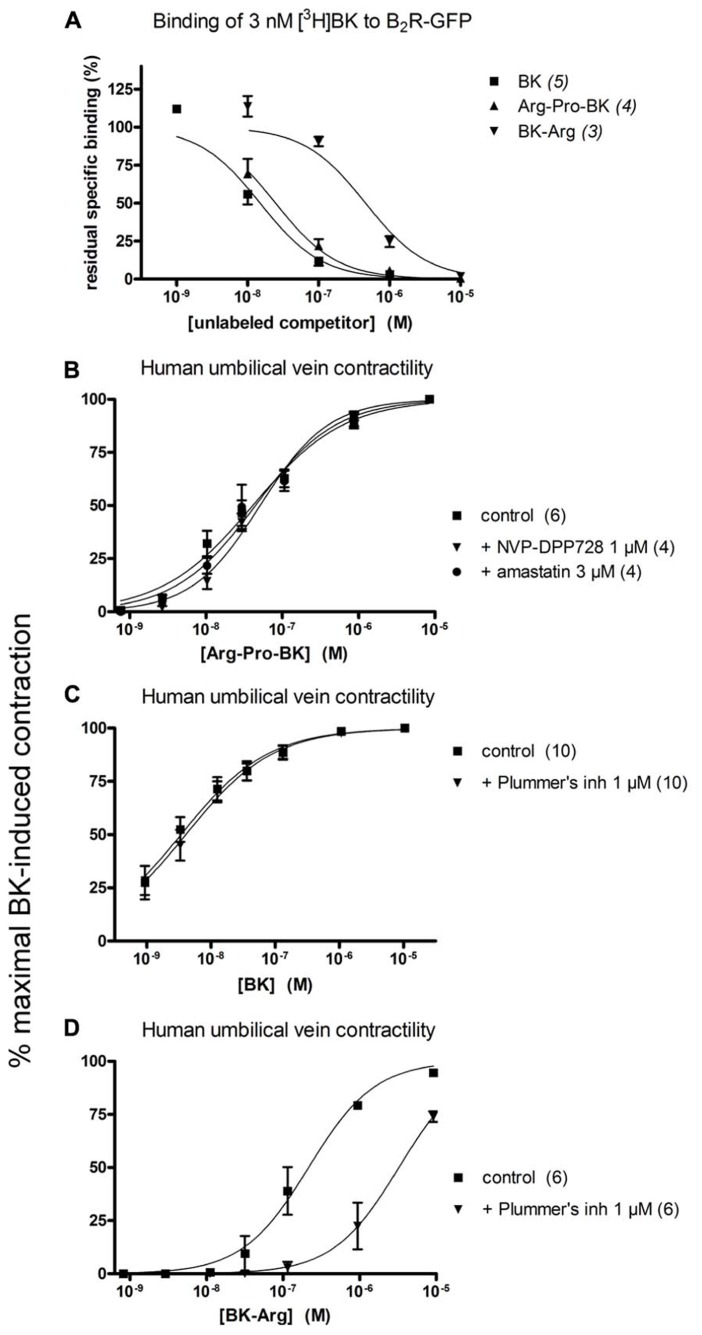
**Pharmacology of Arg-Pro-BK, a potential DPP IV substrate, and of BK-Arg, an arginine carboxypeptidase substate. (A)** Competition of [^3^H]BK (3 nM) binding to stably expressed B_2_R-GFP in HEK 293 cells. **(B)** Cumulative concentration-effect curve for Arg-Pro-BK as modified by the DPP IV inhibitor NVP-DPP728 (1 μM) or the aminopeptidase inhibitor amastatin (3 μM). **(C)** Lack of effect of Plummer’s inhibitor on BK-induced contraction of the human umbilical vein preparation. **(D)** Cumulative concentration-effect curve for BK-Arg as modified by the same drug. Presentation as in **Figures 2** and **3**.

Arg-Pro-BK, designed as a potential substrate of DPP IV, retained a high affinity for the B_2_R (only 1.6-fold less potent than BK; **Figure 5A**; **Table 1**). The umbilical vein contractility assay did not show a loss of apparent potency in the presence of the DPP-IV inhibitor NVP-DPP728 (**Figure 5B**; **Table 2**). Alternatively, BK could be regenerated by two enzymatic cycles catalyzed by aminopeptidases widely expressed in blood vessels, such as aminopeptidase N (Gera et al., 2013b). The aminopeptidase inhibitor amastatin also failed to modify the contractile effect of Arg-Pro-BK (**Figure 5B**; **Table 2**).

**Table 2:** Parameters derived from contractility assays in the human umbilical vein.

Agonist	Data from Figures	Control (DMSO vehicle) log(EC_50_) ± SE	Peptidase inhibitor identity	+Peptidase inhibitor log(EC_50_) ± SE
BK	3A	-7.74 ± 0.07	enalaprilat 1 μM	-7.82 ± 0.09
BK-Ser-Tyr	3B	-6.90 ± 0.09	enalaprilat 1 μM	-6.32 ± 0.06
BK-His-Leu	3C	-6.61 ± 0.11	enalaprilat 1 μM	-5.34 ± 0.06
BK-Ala-Pro	3D	-6.59 ± 0.07	enalaprilat 1 μM	-5.55 ± 0.06
Arg-Pro-BK	5B	-7.39 ± 0.06	NVP-DPP728 1 μM	-7.29 ± 0.03
			amastatin 3 μM	-7.35 ± 0.07
BK	5C	-8.47 ± 0.08	Plummer’s inhibitor 1 μM	-8.39 ± 0.07
BK-Arg	5D	-6.67 ± 0.08	Plummer’s inhibitor 1 μM	-5.49 ± 0.07

BK-Arg had an affinity 29-fold lower than that of BK for B_2_Rs, based on the binding competition of [^3^H]BK (**Figure 5A**; **Table 1**). In the contractility assay, the potency of BK-Arg is reduced 15-fold in the presence of the inhibitor of Arg-CPs, Plummer’s inhibitor. This inhibitor did not affect the potency of BK, given the experimental precision (**Figures 5C,D**; **Table 2**). Together, these observations support the regeneration of BK by endogenous Arg-CP(s).

### AFFINITY OF C-TERMINALLY PROLONGED BK ANALOGS FOR THE KININ B_1_R

**FIGURE 6:**
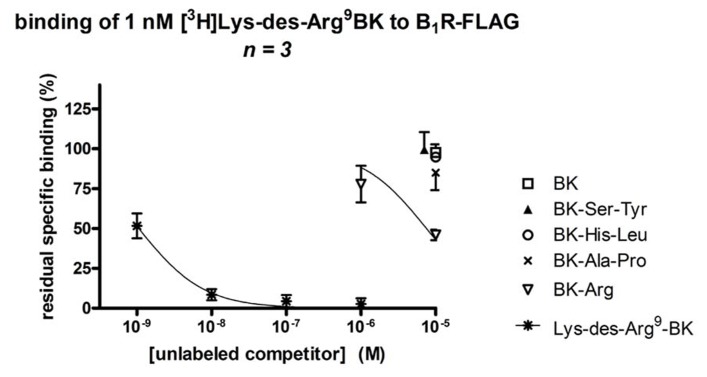
**Competition of [^3^H]Lys-des-Arg^9^-BK (1 nM) binding to transiently expressed human B_1_R (B_1_R-FLAG vector) in HEK 293a cells by C-terminally prolonged BK analogs**. For comparison, unlabeled BK and Lys-des-Arg^9^-BK, the prototypical agonists of the B_2_R and B_1_R, respectively, were also used as competitors. Results are expressed as the residual specific bindings (means ± SE of three duplicate determinations).

Bradykinin itself and the three BK analogs prolonged by a C-terminal dipeptide exhibited a very low affinity (IC_50_ > 10 μM) for the recombinant human B_1_R, as assessed by the binding competition of [^3^H]Lys-des-Arg^9^-BK (**Figure 6**; **Table 1**). Surprisingly, BK-Arg had an affinity for the B_1_R larger than that of BK, but still marginal (IC_50_ = 7.6 μM). The unlabeled form of the radioligand efficiently competed for receptor binding in the nanomolar concentration range.

### EFFECT OF SELECTED C-TERMINALLY EXTENDED BK HOMOLOG ON B_2_R-GFP CYCLING

**FIGURE 7:**
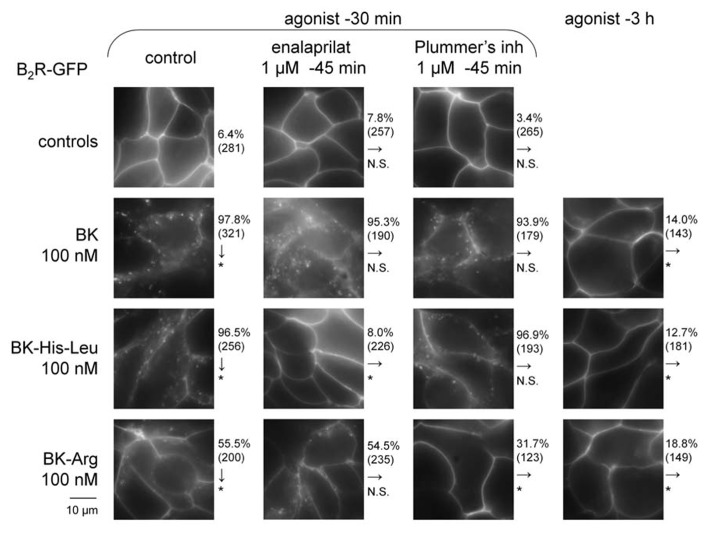
**Effect of BK-related peptides (100 nM) added to the serum-supplemented culture medium of HEK 293 stably expressing B_2_R-GFP on the cycling of this fusion protein**. Green epifluorescence (original magnification 1000×). The effect of co-treatment with peptidase inhibitors is shown for the shorter incubation period (30 min). The effect of each experimental condition on the proportion of cells showing evidence of B_2_R-GFP internalization was evaluated using χ^2^ statistics relative to the control condition indicated by arrows (either control cells for vertical arrows, or the left-most conditions for horizontal arrows; *P < 10^-^^3^, N. S. non-significant).

Human Embryonic Kidney 293 cells stably expressing B_2_R-GFP at the level of their plasma membrane exhibited the known BK-induced internalization of the fluorescent receptor following a 30-min stimulation period in the serum-containing culture medium at 37^°^C (100 nM of BK; **Figure 7**). The cell morphology in green epifluorescence was that of disrupted plasma membrane continuity with abundant and polymorphic cytosolic inclusions. As previously reported, this morphology almost entirely reverted 3 h after BK stimulation, coincident with the disappearance of immunoreactive BK in the culture medium (Bachvarov et al., 2001; Charest-Morin et al., 2013b). This assay was applied to selected C-terminally extended BK homolog that exert, at 100 nM, little or no competition on the binding of [^3^H]BK to B_2_R-GFP: BK-His-Leu (**Figure 2A**) and BK-Arg (**Figure 5A**). After 30 min of treatment with the peptides, the translocation of B_2_R-GFP-associated fluorescence from the plasma membrane to endosomes was moderate for BK-His-Leu, and weaker, but significant for BK-Arg (see χ^2^ statistics in **Figure 7**). However, extensive recycling of the fluorescent receptors to the plasma membrane was also observed at time 3 h post stimulation with the extended BK sequences. While HEK 293 cells do not express ACE unless transfected with the corresponding expression vector (Morissette et al., 2008), their serum-supplemented culture medium contains soluble ACE (Bachvarov et al., 2001) and is also likely to contain soluble Arg-CP activity, at least carboxypeptidase N. The specific inhibitors of these peptidases, enalaprilat and Plummer’s inhibitor, respectively, were applied before 30-min treatment with the BK-related agonists in additional experiments reported in **Figure 7**. While these inhibitors did not influence the very high proportion of cells that exhibited BK-induced internalization of B_2_R-GFP, enalaprilat selectively suppressed that induced by BK-His-Leu and Plummer’s inhibitor significantly abated the effect of BK-Arg (**Figure 7**), consistent with the metabolic activation of the two latter agonists by ACE and an Arg-CP, respectively.

## DISCUSSION

Protease-activated prodrugs would produce little off-target side effects if the distribution of the chosen protease was characteristic of a disease state, e.g., tumors enriched in cathepsins or matrix metalloproteinases (Choi et al., 2012). Exploiting the distribution of ectopeptidases expressed in the vasculature to activate pro-drugs is a novel approach that mimics the natural ACE-mediated gain of function of Ang I. Effective pro-drugs that regenerate BK should have little direct affinity at the B_2_R, which was tested using the [^3^H]BK binding competition assay. Further, BK is the minimal sequence of high affinity at the B_2_R and any fragment (e.g., those generated by ACE) will not be biologically active (Leeb-Lundberg et al., 2005). The current docking model of BK to the B_2_R indicates that the N-terminus of the agonist peptide is close to the extracellular fluid, thus possibly amenable to N-terminal extension, while the C-terminus of BK interacts deep in the receptor central cavity (Leeb-Lundberg et al., 2005), consistent with the more severe loss of affinity for BK sequences prolonged at the C-terminus in the present novel series of peptides. For the latter BK analogs, the gain of function resulting from the regeneration of BK in the venous contractility assay must follow precise cleavage rules. Pharmacologic evidence of ACE-mediated removal of the C-terminal dipeptide of BK-Ser-Tyr, BK-His-Leu and BK-Ala-Pro was obtained as enalaprilat reduced the contractile potency of each of these peptides approximately to the level of its low affinity for B_2_Rs (**Figure 4**). The most favorable design, BK-His-Leu, shares its C-terminal dipeptide sequence with the known ACE substrate Ang I, displaces [^3^H]enalaprilat from recombinant ACE and has an apparent 18-fold gain of function mediated by ACE in the venous contractility assay. Immunohistochemistry of human umbilical vein sections showed that ACE expression is limited to the luminal (endothelial) surface of the vein (Koumbadinga et al., 2010). BK-His-Leu-induced internalization of B_2_R-GFP is selectively suppressed by enalaprilat in HEK 293 cells (**Figure 7**), a system where ACE is supplied by serum-containing culture medium (Bachvarov et al., 2001). In this experimental system, the endocytosis of B_2_R-GFP is largely reversible as a function of time (compare the 30-min stimulation with the 3-h stimulation in **Figure 7**), an effect previously attributed in part to ACE-mediated BK degradation in the culture medium (Bachvarov et al., 2001). However, the very large acute effect of BK on the endocytosis of B_2_R-GFP was not modified by enalaprilat co-treatment.

Only the C-terminal residue must be removed from BK-Arg to regenerate BK and one of the Arg-CPs (kininase I activity) may mediate this; these peptidases include soluble carboxypeptidase N, carboxypeptidase M, and carboxypeptidase D, the two latter being expressed at the surface of human endothelial cells (Sangsree et al., 2003). Carboxypeptidase N assumes a minor pathway of BK degradation in human plasma leading to the formation of des-Arg^9^-BK (Cyr et al., 2001) as BK itself possesses a C-terminal Arg residue. Plummer’s inhibitor (mergetpa), a mercapto analog of Arg (Plummer and Ryan, 1981), blocks Arg-CPs with specificity and reduces the effect of Lys-BK on the rabbit aorta, a contractile bioassay of the kinin B_1_R, because the *in situ* formation of its optimal agonist Lys-des-Arg^9^-BK depends on kininase I (Gera et al., 2011). In the present experiments, a loss of BK-Arg contractile potency in the B_2_R bioassay and of B_2_R-GFP endocytosis in HEK 293 cells in the presence of Plummer’s inhibitor is consistent with Arg-CP-mediated regeneration of BK from BK-Arg in control conditions.

The existence of BK regenerated from either type of C-terminal extended peptides is probably transient in vascular tissue, as the very same activating peptidases also inactivate intact BK. The recycling of B_2_R-GFP to the plasma membrane of cells 3 h after stimulation with either BK-His-Leu or BK-Arg is also consistent with the inherent fragility of the regenerated BK. By contrast, prolonged endocytosis (≥12 h) of B_2_R-GFP is produced in response to several inactivation-resistant B_2_R agonists or partial agonist (Bawolak et al., 2009, 2012). ACE inhibitors exert differential effects in the BK bioassays used in the present study: while captopril increases BK half-life in the culture medium of HEK 293 cells and the duration of B_2_R-GFP endocytosis in these cells (Bachvarov et al., 2001), enalaprilat or captopril failed to potentiate BK in the human umbilical vein contractility assay (**Figure 3A**; Marceau et al., 1994; Gobeil et al., 1996; Bawolak et al., 2007). The latter finding does not automatically apply to all isolated blood vessels, as the BK contractile effect on the rabbit isolated jugular vein was clearly potentiated by enalaprilat treatment (Gera et al., 2011). Previous immunohistochemistry and immunofluorescence studies showed that the umbilical vein possesses a relatively thick media composed of ~30 layers of compactly organized smooth muscle cells (positive for α-actin), while immunoreactive ACE is represented in the single endothelial cell layer (Koumbadinga et al., 2010; Gera et al., 2013b). By comparison, the rabbit jugular vein is very thin, with a higher endothelium/smooth muscle ratio. In the human umbilical vein preparation, due to the low endothelium/muscle ratio, ACE activity may not impair the equilibrium between BK concentration in the bathing fluid and that at the vicinity of most venous muscle cells (the general problem of non-equilibrium drug distribution in isolated vascular tissue is discussed by Marceau et al., 2010). However, ACE presence in the umbilical vein is functionally revealed by the metabolic activation of prodrug peptides that regenerate BK.

We have previously explored several N-terminal extensions of kinin sequences to produce conjugates with fluorophores, drug-like molecules and an antigenic tag (Gera et al., 2012, 2013a). These agents were generally afflicted by a severe loss of affinity (2–3 log units), although other N-terminally extended sequences (Met-Lys-BK, maximakinin, the GFP-maximakinin fusion protein) retain excellent affinities at the B_2_R (Bawolak et al., 2012; Charest-Morin et al., 2013a). This was also the case for the novel analog Arg-Pro-BK, designed as a DPP IV substrate (**Figure 1**). The conserved affinity of Arg-Pro-BK for the B_2_R precludes the observation of an activation reaction in the venous contractility system. However, the latter may contain such aminopeptidases: we have recently observed that L-Ala-histamine-induced contraction of the isolated umbilical vein largely results from the regeneration of free histamine by aminopeptidase(s) sensitive to amastatin (Gera et al., 2013b), thus extending the concept of vasopeptidase-activated prodrug to another receptor system (the histamine H_1_ receptor).

Current evidence favors the preformed B_2_R over the inducible B_1_R as the pharmacological entity that mediates most cardiovascular benefits of kinins (see Introduction). The novel C-terminally extended BK analogs have little or no direct affinity for the B_1_R (**Figure 6**) and are not likely to release significant amounts of a human B_1_R agonist for the following reasons: (1) the optimal agonist of the human form of the B_1_R is Lys-des-Arg^9^-BK, not des-Arg^9^-BK which has little affinity (Leeb-Lundberg et al., 2005). The designed prodrug peptides are all based on BK, not Lys-BK, and cannot generate Lys-des-Arg^9^-BK. (2) It is not excluded that they indirectly produce small amounts of des-Arg^9^-BK from regenerated BK in the umbilical vein. However, authentic BK is competitively antagonized by various B_2_R antagonists in this bioassay, not by a selective B_1_R antagonist (Marceau et al., 1994; Gobeil et al., 1996; Bawolak and Marceau, 2007), showing that des-Arg^9^-BK, if generated from BK, does not reach pharmacologically active concentrations. (3) B_1_R-mediated contraction in the human umbilical vein starts from a low maximal effect that increases as a function of the incubation time, being weak 2 h post-tissue mounting and more intense at the time point 5 h (Houle et al., 2000). By contrast, the contractile effect mediated by the B_2_R is stable (2–6 h; Marceau et al., 1994). Thus any contribution of the B_1_R to the effects of C-terminally extended BKs is unlikely in the umbilical vein assay as applied.

BK-His-Leu and BK-Arg are examples of pro-drug B_2_R agonist peptides activated by peptidases expressed in vascular tissue and blood plasma. Thus, novel peptides extended around the BK sequence behave as peptidase-activated B_2_R agonists that may be selective for the vascular system and support further investigation of the cardiovascular benefits of kinins.

## Conflict of Interest Statement

The authors declare that the research was conducted in the absence of any commercial or financial relationships that could be construed as a potential conflict of interest.
